# The mechanism underlying inhibition of saccadic return

**DOI:** 10.1016/j.cogpsych.2009.04.002

**Published:** 2009-09

**Authors:** Casimir J.H. Ludwig, Simon Farrell, Lucy A. Ellis, Iain D. Gilchrist

**Affiliations:** University of Bristol, Department of Experimental Psychology, 12a Priory Road, Bristol BS8 1TU, UK

**Keywords:** Inhibition of return, Saccadic eye movements, Computational model, Temporal integration, Reaction time, Latency distribution

## Abstract

Human observers take longer to re-direct gaze to a previously fixated location. Although there has been some exploration of the characteristics of inhibition of saccadic return (ISR), the exact mechanisms by which ISR operates are currently unknown. In the framework of accumulation models of response times, in which evidence is integrated over time to a response threshold, ISR could reflect a reduction in the rate of accumulation for saccades to return locations or an increase in the effective criterion for response. In two experiments, participants generated sequences of three saccades, in response to a peripheral or a central cue. ISR occurred across these manipulations: saccade latency was consistently increased for movements to the immediately previously fixated location. Latency distributions from individual observers were fit with a Linear Ballistic Accumulator model. ISR was best accounted for as a change in the accumulation rate. We suggest this parameter represents the overall desirability of a particular course of action, the evidence for which may be derived from a variety of sensory and non-sensory sources.

## 1 Introduction

Visual attention is generally thought of as a gating mechanism that facilitates the processing of a relevant subset of visual signals (Broadbent, 1958). Visual processing is typically enhanced at an attended location, resulting in shorter detection times and/or improved discrimination accuracy relative to non-attended locations (Muller & Findlay, 1987; Posner, 1980; Yeshurun & Carrasco, 1998). In the classic cueing paradigm a salient cue is presented to attract attention, and perceptual performance is assessed for a subsequent stimulus presented either at the cued location or at an uncued location. This sequence of events is thought to involve three “movements” of attention: first to the cue, then back to the central fixation point, and finally to the target stimulus. The well-established finding is that when the interval between cue and stimulus is short, performance is improved at the cued location (Klein, 2000; Posner & Cohen, 1984; Samuel & Kat, 2003). However, for longer intervals performance is actually worse. This latter phenomenon has been interpreted as a delay in the re-allocation of attention to the already visited location, and has been labelled ‘Inhibition of Return’ (IOR) accordingly. Functionally, IOR has been suggested to promote the efficient exploration of space by preventing an observer from returning to previously attended locations: that is, IOR is seen as a ‘foraging facilitator’ in the search for information (Klein, 2000; Klein & MacInnes, 1999).

Given the close coupling between covert attention and saccadic eye movements (Deubel & Schneider, 1996; Kowler, Anderson, Dosher, & Blaser, 1995; Kustov & Robinson, 1996), it is not surprising that IOR has also been found with saccadic responses (Rafal, Egly, & Rhodes, 1994; Taylor & Klein, 2000; Vaughan, 1984). Indeed, if IOR has a role to play in promoting efficient visual exploration it should manifest itself in tasks that involve active exploration through sequences of saccades. In particular, observers should be biased against returning to a previously fixated location (Gilchrist & Harvey, 2000; McCarley, Wang, Kramer, Irwin, & Peterson, 2003; Peterson, Kramer, Wang, Irwin, & McCarley, 2001). Klein and MacInnes (1999) presented evidence from a visual search task showing that the time taken to initiate saccadic eye movements to previously fixated locations was longest for those visited very recently (specifically, two fixations back). Slowing in return saccades has also been seen under more controlled conditions in which saccade sequences are cued or instructed. In a study examining sequential effects in saccade initiation, Hooge and Frens (2000) had observers scan around a set of dots that were arranged in a variety of configurations across conditions. For example, in one of the experiments the observer fixated a left, central, and a right dot sequentially before a reversal in direction (i.e., from right to left, and so on). The scanning was self-paced, with the relevant measure being the fixation duration on a given dot prior to a movement to the next location. These fixation durations were indeed consistently prolonged before a return saccade from one of the outer dots to the central dot. In other experiments, Hooge and Frens showed that this effect was not simply due to a general “direction-reversal”, but that the increase in latency was spatially specific (<4°) for the previously fixated location (see also Vaughan, 1984). A similar gradient of inhibition has also been found using more standard cueing paradigms and manual responses (Bennett & Pratt, 2001; Klein, Christie, & Morris, 2005). In a more typical visually guided saccade task Carpenter (2001) found that observers were slower to respond to a target that appeared in the same location as on the previous trial. Taking the intervening return saccade into account, it may be that the saccadic system has a preference for moving in the same direction (Anderson, Yadav, & Carpenter, 2008).

This and other evidence (Findlay & Brown, 2006; Hooge, Over, van Wezel, & Frens, 2005) suggests that human observers are biased against moving the eyes to a previously fixated location. We refer to this effect as Inhibition of Saccade Return (ISR; Hooge & Frens, 2000), although we acknowledge that ISR and IOR may well be manifestations of the same underlying mechanisms. ISR appears to be a general phenomenon in oculomotor control, occurring in visual search (Gilchrist & Harvey, 2000; Hooge et al., 2005), in performance of pre-determined saccades sequences (Findlay & Brown, 2006; Hooge & Frens, 2000) and for visually guided saccades (Carpenter, 2001; Klein & MacInnes, 1999). However, previous research has generally focused on determining the characteristics of the phenomenon (e.g., its spatial tuning), and a precise mechanism by which the eyes are biased against returning to previously fixated locations has not been described. Accordingly, in this paper we consider how ISR might be accounted for in existing models of saccadic and perceptual decision making. We show that models of evidence accumulation allow us to delineate some possible mechanisms that underlie ISR.

### 1.1 Models of evidence accumulation

Saccade generation, like simple perceptual decisions in manual reaction time studies, can be effectively modelled as a process of accumulating evidence up to some criterion (Carpenter & Williams, 1995; Hanes & Schall, 1996; Ludwig, Mildinhall, & Gilchrist, 2007; Ratcliff, Cherian, & Segraves, 2003). A variety of such models exist that differ in their specific assumptions, but they share the common idea that a response is generated as soon as an integrated quantity reaches some threshold value (Brown & Heathcote, 2005, 2008; LaBerge, 1962; Palmer, Huk, & Shadlen, 2005; Ratcliff & Smith, 2004; Usher & McClelland, 2001; for reviews, see Luce, 1986; Smith & Ratcliff, 2004). Variability in (saccade) latency is typically accounted for by assuming that the accumulation rate varies from moment to moment (e.g., Ratcliff & Rouder, 1998; Ratcliff & Smith, 2004) and/or between responses (e.g., Brown & Heathcote, 2005, 2008; Carpenter, 1981). Many models also incorporate variability in the starting point of accumulation (Brown & Heathcote, 2008; Ratcliff & Rouder, 1998).

**Fig. 1:**
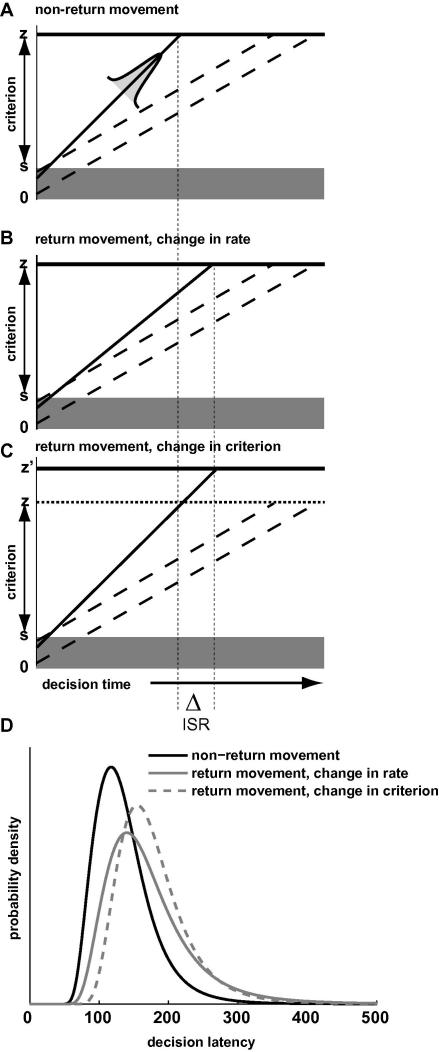
LBA account of ISR. (A) Race to threshold between three independent accumulators, each subject to Gaussian variability in the accumulation rate and uniform variability in the starting point of accumulation. The accumulator coding the target location is shown by the solid line. The competing non-target locations are coded by the dashed accumulators. (B) The target is at a return location, causing a reduction in the accumulation rate of the associated movement programme. As a result, the threshold is crosses at a later point. (C) The target is at a return location, resulting in a selective increase in the evidence criterion for the accumulator coding the target and a delay in the time at which threshold is reached. (D) Corresponding predictions of the latency distributions when all other parameters (starting point and rate variability, non-target accumulation rates) are kept constant.

A conceptually simple and mathematically tractable model for choice and reaction time is the Linear Ballistic Accumulator model (Brown & Heathcote, 2008). This model assumes a linear, noise-free accumulation to criterion, with the rate of accumulation varying between responses in a Gaussian manner and a uniform distribution of starting points. Each response alternative is associated with its own, independent accumulator. Multiple accumulators compete with each other in a race to threshold. The response associated with the winning accumulator is executed, with a latency determined by the time at which the threshold was reached. This model is illustrated in the panel A of Fig. 1, which shows what may be regarded as the ‘decision period’ of a fixation period prior to movement initiation. In line with other accumulator models we also assume an additional, constant non-decisional latency that includes afferent and efferent delays associated with stimulus encoding and peripheral motor delays (Brown & Heathcote, 2005; Ratcliff & Rouder, 1998).

The figure shows the amount of accumulated evidence as a function of time for a number of independent accumulators. The accumulator indicated by the solid line is the one associated with the desired target-directed response. The remaining accumulators are associated with non-target (error) responses. Each accumulator starts at a random point over the interval [0, *s*] and rises towards threshold *z*. The accumulation rate varies from trial to trial (or, more appropriately, for each saccade) according to a Gaussian distribution. Together these assumptions of variability in starting point and accumulation rate are sufficient to produce latency distributions that have the appropriate typical right-skewed shape (see panel D of Fig. 1). Brown and Heathcote (2008) demonstrated that the model provides a reasonable description of choice and response time data from a range of different psychological paradigms.

The appeal of models such as LBA is their link with the underlying neural mechanisms of decision making, particularly in the case of saccadic choice. Neural integrators that, in at least some aspects, resemble those assumed by evidence accumulation models have been found in a variety of brain structures that are involved in saccade target selection such as the superior colliculus (SC; Ratcliff et al., 2003), frontal eye fields (FEF; Hanes & Schall, 1996), and lateral intraparietal area (LIP; Gold & Shadlen, 2003; Roitman & Shadlen, 2002). Subsets of neurons in these areas gradually increase their firing rate over time, and the rate of increase is predictive of the latency of the saccadic response (Hanes & Schall, 1996; Roitman & Shadlen, 2002). In addition, when the neural activity is aligned on saccade onset it appears that a constant amount of accumulated activity is associated with saccade initiation (Hanes & Schall, 1996; Roitman & Shadlen, 2002). Thus, a single cell may vary in its build-up rate from trial to trial, but the amount of activity needed to initiate a response is invariant to this rate (Ratcliff et al., 2003).

Critically, these neurons respond to various experimental manipulations in a similar manner to that which would be expected from models derived from behavioural data. For example, the effect of the prior probability of a response alternative in a choice task affects the shape of the distribution in a way associated with a change in the starting point for an accumulator (Carpenter & Williams, 1995). Build up neurons in SC (Basso & Wurtz, 1997, 1998; Dorris & Munoz, 1998) as well as LIP neurons (Churchland, Kiani, & Shadlen, 2008) show a similar sensitivity to prior probabilities by adjusting their resting baseline of activation. In addition, the assumption that stimulus quality (i.e., the amount of information that can be extracted from the stimulus per unit of time) will affect accumulation rate (Ratcliff & Rouder, 1998; Reddi, Asrress, & Carpenter, 2003) is consistent with the observation that varying the information content of a stimulus (e.g., the signal-to-noise ratio in a motion coherence stimulus) changes the rate of accumulation in LIP neurons (Roitman & Shadlen, 2002).

With regard to ISR, there are two obvious candidate mechanisms in these models to produce an overall (e.g., mean or median) latency difference between saccades to a previously fixated location and those to a non-return location. First, it may be that the mean rate of accumulation differs so that it takes longer to reach criterion for return saccades compared to non-return movements. This mechanism is illustrated in Fig. 1B. The accumulators associated with the non-targets have the same rate as in the panel above, but the target-related movement programme rises at a lower rate. It still manages to win the race to threshold, but compared to the panel above the latency at which the threshold is reached is delayed (see the shift in the vertical dotted line). Second, it is possible that the mean accumulation rate is the same for both types of saccades, but that more evidence is required specifically for a return movement. This corresponds to either a decrease in the mean starting point of accumulation or an increase in the threshold (these are formally equivalent and cannot be distinguished in the model). For mathematical and conceptual convenience we illustrate this mechanism as a change in threshold in Fig. 1C. It should be noted that the critical variable is the difference between the mean starting point and response threshold, given by *z* − *s*/2. We refer to this variable throughout as the evidence criterion. Panel C shows three accumulators that rise to threshold at the same rates as in panel A, but the threshold associated with the return movement programme is selectively raised (to *z*′) to increase the criterion amount of evidence associated with a return saccade. As a result, compared to panel A it takes longer for this movement to be initiated.

Coupled with the two sources of variability, the latency distributions corresponding to the conditions sketched in panels A – C are shown in panel D. Clearly, both a decrease in the mean accumulation rate and an increase in the evidence criterion results in an overall (mean or median) inhibitory effect. That is, both return movement distributions lie to the right of the non-return distribution. However, the two different mechanisms make different predictions about the width and shape of the distributions. Thus, only through consideration of the *entire* distribution can the underlying mechanism that caused an overall latency difference be identified (Van Zandt, 2000).

The aim of our study was to identify the underlying mechanism(s) of ISR. As demonstrated in Fig. 1, we can use process models to go beyond simply observing a latency difference to making plausible inferences as to *why* that latency difference occurs. More specifically, ISR may correspond to a reduction in the accumulation rate, an increase in the evidence criterion, or both. These are both plausible candidate mechanisms. It is reasonable to regard ISR as a prior assumption that recently inspected locations are unlikely to become behaviourally relevant in the next instant. Such a modification of the prior probability is most consistent with a change in the evidence criterion (Carpenter & Williams, 1995). Then again, it is possible that ISR reflects a slowing in the extraction of information from the stimulus. This is perhaps more consistent with the notion of the inhibitory effect having an attentional or perceptual locus (Taylor & Klein, 1998), an issue to which we return in the Section 5. Our strategy to tease these possibilities apart was to fit the latency distributions of saccades to return and non-return locations with a model in which differences between these distributions (i.e., ISR) could be accounted for by both mechanisms. Subsequent examination of the rate and criterion parameters can then identify the relative contributions of the two mechanisms to the ISR observed behaviourally.

We devised a gaze-contingent experimental paradigm in which human observers made sequences of three saccadic eye movements. After each saccade observers were cued as to where to look next. This allowed tight experimental control over where and when the observer should look. This paradigm enabled us to create a number of different movement sequences that involved return saccades on the second saccade, third saccade, both, or no return saccades at all. In addition, a sequence of three movements allowed for the possibility of having observers look to a location they fixated two movements in the past. In this way, we can look for ISR in 1-back and 2-back saccades. Most importantly, the movement paths were designed to enable comparison between return saccades and those to a non-return location, uncontaminated by the preceding movements in the sequence (see below). This paradigm is essentially an attempt to combine the sequential nature of a scanning task such as visual search or picture viewing, with the tight experimental control afforded by simple visually guided saccade tasks or cueing paradigms. The task shares similarities with the gaze-contingent visual search task used by McCarley et al. (2003), with the critical difference that in their task observers were free to decide which of two possible items to fixate. Across two experiments we varied the nature of the cue that signalled to observers where to look next.

## 2 General methods

### 2.1 Observers

Six observers took part in each experiment (across both experiments: seven female observers, age range 20–27 years, all with normal or corrected-to-normal vision). The experimental procedures were reviewed by the local Human Research Ethics Committee. Some observers took part for course credits, others (particularly in Experiment 2) received payment. Experiment 1 was completed in a single 1-h testing session for each observer. Experiment 2 involved six 1-h sessions. All observers in both experiments had normal or corrected-to-normal vision.

### 2.2 Stimuli

**Fig. 2:**
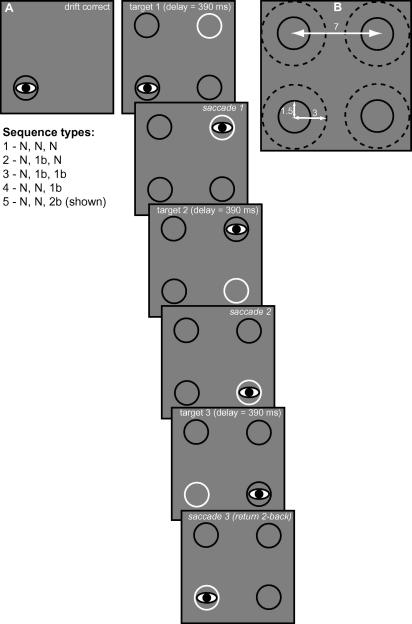
Stimulus design in Experiment 1. (A) Sequence of events over the course of a “trial” of three saccades. At the beginning of each saccade sequence a drift correction occurred at one of the four possible starting locations. After a fixed interval the remaining display elements appeared and the saccade sequence started by moving the eyes to the white target ring. Subsequent display changes were contingent upon the eye reaching the target ring within a certain critical distance with a single movement. The five possible sequence types are listed: ‘N’ refers to a location not previously fixated within the current sequence; ‘1b’ refers to the location fixated before the immediately preceding saccade; ‘2b’ refers to the location fixated before the previous two saccades (i.e., at the very start of the sequence). The illustrated sequence is of ‘type 5’. (B) Display parameters. The dashed circle illustrates the invisible critical region around each ring.

Target displays consisted of four rings, one of which was fixated by the observer, one of which was the target of the next saccade, and two that were non-targets. The rings were presented on a grey background with a luminance of 52.4 cd/m^2^. Fig. 2 shows the lay-out of the display. The rings had radii of 1.5° and were presented in square configuration in the centre of the screen, with a centre to centre separation between adjacent rings of 7° (making the diagonal distance almost 10°). An invisible ‘acceptance region’ surrounded each ring (indicated by the dashed circles in Fig. 2B). The radius of this window was 3° and it served to classify saccades online. Observers viewed the stimuli in a darkened room on a 21 in. CRT monitor (EIZO Flexscan T965, 1152 × 864, running at 75 Hz) at 57 cm with their head resting on a chin rest.

### 2.3 Eye movement recording

The EyeLink II (SR Research Ltd.) system was used to record eye movement data from observers’ dominant eye. This system tracks the centre of the pupil with a spatial resolution of ∼0.3° and a sampling rate of 500 Hz. Saccades were classified using velocity and acceleration criteria of 30°/s and 8000°/s^2^, respectively. The 2-D eye position was used online to monitor the accuracy of the ongoing saccade sequence, and to control the timing of target presentation.

### 2.4 Procedure

At the start of each sequence the observer was asked to fixate the centre of a single dark ring presented on the monitor (see Fig. 2A, ‘drift correct’). Upon accurate fixation three additional rings appeared on the screen after a delay. One of these three rings was the target for the first saccade (Fig. 2A, ‘target 1’ illustrates how the target was cued by the brightness of the ring in Experiment 1). Observers were asked to fixate the centre of the brighter ring with a single saccade. If the saccade was accurate according to the criteria outlined below (Fig. 2A, ‘saccade 1’) a response–stimulus interval of approximately 390 ms was imposed, after which the next target was presented. After a successful second saccade, the third saccade was cued in the same manner.

For each saccade the start and landing positions were compared to the co-ordinates of the current fixation and target rings. Smaller movements within the fixation ring (within the 3° critical window) were not classified as orienting movements; such corrective movements are a ubiquitous component of saccadic orienting and prolonged fixation (Carpenter, 1988). However, once a larger movement was generated that took gaze outside the current fixation region, the algorithm checked whether the landing position was in the acceptance region surrounding the target ring. If so, the subsequent target was presented. If a saccade landed outside the acceptance region, the sequence was stopped and a new sequence was initiated. The erroneous sequence was repeated at the end of the block. The new sequence started with the presentation of a single dark ring. This cycle was continued until a criterion number of 60 accurate sequences was obtained within a block. Observers completed five blocks in an experimental session.

Given the square configuration and sequences of three saccades, five different sequence types can be distinguished on the basis of (a) whether the second saccade takes the observer back to the location of the immediately preceding fixation (1-back return saccade) or to a location that has not yet been fixated (non-return) and (b) whether the third saccade was a 1-back return saccade, a 2-back return saccade (shown in Fig. 2A – the third saccade takes the observer back to the original starting position), or a saccade to a non-return location. These different sequences are listed in the inset of Fig. 2. Each of the 12 possible combinations of four starting locations and three first target locations was presented five times in a block of 60 sequences. The second and third target locations depended on the exact sequence type. For instance, once the starting and first target locations have been assigned, the remainder of the sequence is fully determined in case of sequence type 3 (non-return, 1-back, 1-back; see Fig. 2). However, for sequence types 1, 4 and 5 the first two targets are non-return. For these sequences the second target is randomly chosen from the two remaining non-return locations, after which the third target location is fully determined. Finally, for sequence type 2 the second target is fixed, after which there are two remaining possible non-return target locations. The target for the third saccade was randomly picked from these two options. All sequence types were repeated equally often in a block.

Of critical interest is the comparison between 1-back and non-return for second saccades (sequence types 2 and 3 combined versus 1, 4 and 5 combined), between 1-back and non-return for third saccades (sequence types 4 versus 1), and between 2-back and non-return for third saccades (sequence types 5 versus 1). Note that to evaluate ISR for second saccades we combined the data from sequence types 2 and 3 (for return saccades) and from 1, 4, and 5 (for non-return saccades), because the sequences within a group are identical up to the second saccade. For the third saccades we only analysed data from sequence types 1, 4, and 5. Again, these sequences are identical up to the third saccade. Therefore, any ISR effects, whether a 1- or 2-back effect, cannot be attributed to differences in the movement path prior to the saccade of interest.

### 2.5 Model fitting

Saccade latency distributions were first de-trended to remove the effects of saccade direction. It is well-established that downward saccades tend to have longer latencies than upward saccades (Honda & Findlay, 1992; Ludwig & Gilchrist, 2003). In addition, observers often show idiosyncratic left-right saccade latency differences. Finally, the amplitude of diagonal saccades in this design was clearly larger than that of pure horizontal and vertical movements. Although we would not expect latency differences between 7° and 10° amplitude saccades (Kalesnykas & Hallett, 1994), we removed these directional sources of variability that might otherwise mask subtle differences in the location and shape of the latency distributions of interest. De-trending was achieved by aligning the mean of each individual “directional” distribution with the overall, grand mean latency, for each observer.

The critical model parameters are the mean accumulation rates for the target and non-targets (*v_T_* and *v_NT_*, respectively), the upper limit of the uniform starting point variability (*s*), the response threshold (*z*), and the accumulation rate standard deviation (*σ_v_*). For a given vector of parameters, the predicted probability density for the target-related decision times is given by Eqs. (1)–(3) in Brown and Heathcote (2008) (for the sake of brevity, these equations are not reproduced here). Note that the model predicts *defective* probability densities, that is, densities that integrate to the probability of the associated response (which will be below 1 if errors occurred). Two additional parameters were introduced to accommodate a difference between a return and non-return latency distribution. These took the form of multipliers that enabled selective modification of the mean accumulation rate and evidence criterion associated with return locations. That is, the accumulator corresponding to a return location had a mean accumulation rate of *αv* and a threshold of *βz*. The parameters *α* and *β* therefore quantify the effect of previous fixation on the accumulation rate and evidence criterion. Note that the return location equates to the target location in the case of a return saccade, but to a non-target location for non-return saccades. Finally, the constant non-decisional delay (*T_er_*) brought the total number of parameters to eight. All parameters, along with a brief explanation, are listed in Table 1.

It is customary to fix one of these parameters to an arbitrary constant, as it sets the scale of the model. In the model fits reported here the basic, non-return response threshold was fixed to 1. This leaves a total of seven free parameters that were varied to account for two whole latency distributions. The observed latency distributions for correct responses were binned into six quantiles, defined by the following boundaries: {0.1, 0.3, 0.5, 0.7, 0.9} (Ratcliff & Smith, 2004). A seventh bin contained the proportion of erroneous responses, namely those saccades that left the currently fixated quadrant, but did not land inside the target acceptance region. For a given set of model parameters, we computed the predicted proportion of responses falling within each of the six latency bins. The predicted error rate is given by 1 minus the total area under the predicted defective probability density (i.e., the integral of Eq. (3) in Brown & Heathcote, 2008). This calculation was performed separately for the case where the target was at a return location and when the target was at a non-return location. Across the pair of distributions we computed the likelihood ratio chi-square measure of goodness-of-fit:

(1) $$G^{2}=2\overset{14}{\underset{i=1}{\sum }}{\mathrm{Np}}_{i}\ln\left[\frac{p_{i}}{\pi_{i}}\right]\text{,}$$

where *i* = 1 … 14 indexes the 14 bins across both distributions, *N* is the total number of trials, *p_i_* is the observed proportion of responses in bin *i*, and *π_i_* is the model prediction for that bin (Ratcliff & Smith, 2004). The *G*^2^ was minimized using the Simplex search algorithm (Nelder & Mead, 1965). The algorithm was initiated with 16 different sets of starting parameters, chosen on a regular grid. We then selected the best fit from these 16 runs. In this way we could be confident that the best fit indeed represented a global minimum.

## 3 Experiment 1

In this experiment the movement cue was presented in peripheral vision. For such cues there is a direct mapping between the stimulus and response. Saccades triggered under such conditions are sometimes called exogenous or even reflexive (Walker, Walker, Husain, & Kennard, 2000). We avoid this terminology by describing the nature of the cue rather than that of the saccade. Our view is that all saccades are endogenous, as in principle participants can always choose to suppress saccades altogether in this paradigm regardless of the visual events on the screen. The events in a single sequence are illustrated in Fig. 2. The brightening of one of the three peripheral rings served as the target cue. The brightness difference was fairly subtle: 56.3 cd/m^2^ (target luminance) versus 48.5 cd/m^2^ (non-target luminance). However, the rings were clearly visible against the background and observers appeared to have no difficulty making the correct target discrimination, as evidenced by low error rates (see Table 2).

### 3.1 Results

Across observers the number of valid sequences ranged from 207 to 300 (after excluding anticipatory saccades with latencies below 80 ms and blocks that were terminated prematurely due to eye-tracker failure). This group of responses itself is distributed across the five sequence types, so that the number of included saccades for any one type of sequence ranged from 39 to 60.

**Fig. 3:**
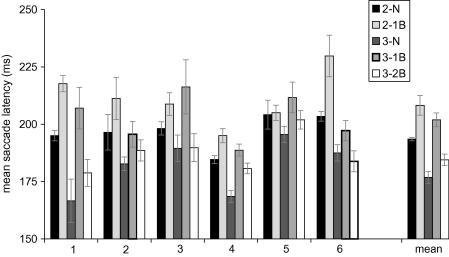
Mean saccade latency for individual observers and the average across observers in Experiment 1. Bar labelling is as follows: 2-N = second saccade to a non-return target (sequences 1, 4, and 5 combined); 2-1B = second saccade back to the previously fixated location (sequences 2 and 3 combined); 3-N = third saccade to a non-return target (sequence 1); 3-1B = third saccade back to the previously fixated location (sequence 4); 3-2B = third saccade back to the location fixated before the previous two saccades (sequence 5). Error bars are ±1 SEM (the error bars around the population average are within-subject SEM).

Fig. 3 shows the mean saccade latencies for individual observers, for each of the conditions of interest. The final set of bars in this chart shows the averaged mean latencies from the six observers. This overall pattern is highly representative of that of individual observers (with the occasional exception; e.g., the absence of a 1-back effect for the second saccades of observer 5). A number of strong trends emerge. First, an average ISR effect is present for second saccades (∼14 ms; compare black and light grey bars) and third saccades (∼21 ms; compare dark with medium grey bars). Second, a 2-back effect may be present for some observers (e.g., observer 4), but overall appears much smaller than the 1-back effect. Finally, third saccades of the sequence have an overall tendency to shorter latencies.

A repeated-measures ANOVA was performed to assess the reliability of the 1-back effect across both second and third saccades. The main effect of return versus non-return was highly significant, *F*(1, 5) = 31.29, *p* < .01, as was the main effect of saccade number, *F*(1, 5) = 31.29, *p* < .05. There was no interaction between these two factors, suggesting that ISR was independent of saccade number. A separate paired comparison of the 2-back effect (2-back versus non-return) was marginally significant, *t*(5) = 4.59, *p* = .09. Considering saccade accuracy, the average proportion of saccades that landed within the target acceptance region is listed in Table 2. It is clear that performance is close to ceiling (>90% correct) and does not appear to be affected very much by the return manipulation.

**Fig. 4:**
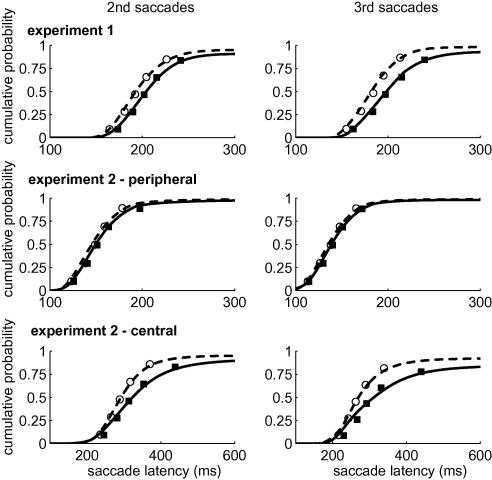
Cumulative probability distributions of 1-back return and non-return saccade latencies, separately for second (left) and third (right) saccades. Each row corresponds to one experimental condition tested in the study. Note that these averaged distribution functions are presented for illustration purposes only; the model fits were performed for each observer individually.

Given a small and unreliable 2-back effect, the modelling focused on 1-back ISR, separately for second and third saccades. The top row of Fig. 4 illustrates the average model fits. The data points correspond to the observed cumulative probability distribution. To construct these plots, each individual observer’s distributions were evaluated at the same quantile boundaries used for model fitting. The saccade quantiles were then averaged across observers to compute vincentised cumulative distribution functions (Ratcliff, 1979). The smooth curves show the average model fits, obtained by averaging the cumulative densities across observers. Note that the fits were done *for each individual observer separately*; we averaged the observed and model quantile distributions in Fig. 4 merely for illustration purposes. As stated earlier, LBA predicts defective probability densities. As such, the level of the upper asymptote of the cumulative distribution function corresponds to the predicted accuracy. Table 2 lists the difference between the observed proportion correct and that for the model (in parentheses). It is clear that the model accuracy generally lies close to that observed in the data and, more importantly, that the model naturally predicts a small effect of ISR on accuracy (i.e., the solid line asymptotes below the dashed function in Fig. 4). The model is able to handle both latency and accuracy data very well. As such, we have solid grounds to examine the parameter estimates to identify the underlying mechanism of ISR.

**Fig. 5:**
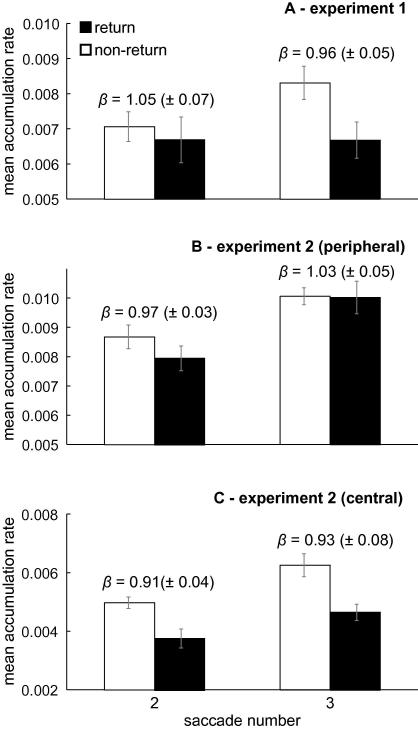
Mean target accumulation rates across observers for return and non-return saccades in Experiment 1 (panel A), the peripheral condition of Experiment 2 (panel B), and the central condition of Experiment 2 (panel C). The rate estimates are derived from the fits to the 1-back data from second and third saccades. For each of these effects the estimated return threshold is shown by the value of parameter *β*. Error bars (and numbers in parentheses) are within-subject SEM.

Fig. 5A shows the mean accumulation rates estimated in the model fits; mean rates are plotted separately for second and third saccades in the left and right columns, respectively. In addition, the average return threshold (±1 SEM) is given in text above the bars. Note that the non-return threshold was set to 1 so that the threshold multiplier *β* simply equates to the return threshold. If ISR is mediated by a change in the underlying rate of accumulation, the rate should be lower for return saccades compared to non-return saccades. If ISR reflects the need for more evidence, the threshold should be elevated for return saccades. The mean accumulation rate for return saccades is, on average, lower than that for non-return saccades. An ANOVA with saccade number and target location (return versus non-return) as factors resulted in a marginally significant effect of target location, *F*(1, 5) = 6.12, *p* = .06. Examining the rates separately for second and third saccades shows that this effect is largely mediated by the third saccade data; indeed the interaction between target location and saccade number was marginally significant, *F*(1, 5) = 5.08, *p* = .07. The return thresholds fell within one standard error of that of the non-return threshold, demonstrating that the evidence criterion remained fixed.

### 3.2 Discussion

A reliable 1-back effect was observed for both second and third saccades. The 2-back effect was much smaller in magnitude and not reliable, and so the 1-back ISR effect was the focus of our modelling. The model allows for the effect to be accommodated through a lower accumulation rate, an increased evidence criterion for return saccades, or both. The model fits the data well and suggests that the critical mechanism is a change in the accumulation rate, particularly for third saccades. The evidence in favour of this mechanism was more mixed for second saccades. However, it should be noted that the second saccade ISR effect was relatively small, and that the potential to find a difference in model parameters will be directly limited by the size of the empirical effect. In addition, the distributions themselves contained a relatively small number of trials and the use of a quantile fitting method implies the inevitable loss of potentially diagnostic information. Concerns about the reliability of parameter identification are reasonable under such conditions.

**Fig. A1:**
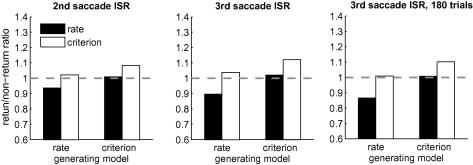
Parameter identification simulation. The values of the proportional scaling parameters *α* (for the return accumulation rate) and *β* (for the return threshold) are shown, depending on which nested model generated the data. The horizontal dashed lines indicate the expected parameter values if a particular mechanism played no causal role in ISR. The bars correspond to the means over 600 simulated and fitted data sets.

One potential objection we address at this stage is that changes in the model parameters are isomorphically linked to changes in the underling mechanisms. That is, we assume that if the “true” underlying model that generated the data involved a change in the accumulation rate and/or criterion parameters, that this will be reflected in the corresponding parameters of the model fits. However, this may not necessarily be the case. For instance, it may be that one mechanism (say, a change in rate) is much better at accounting for data that was, in fact, generated by the alternative mechanism (say, criterion) than vice versa (essentially an asymmetry in model mimicry; cf. Wagenmakers, Ratcliff, Gomez, & Iverson, 2004). To assess the extent to which changes in mechanisms will be accurately reflected in changes in model parameters, we performed a benchmarking simulation. In this simulation we generated data either assuming a change in accumulation rate with a constant criterion (“rate model”), or a change in evidence criterion with a constant mean accumulation rate (“criterion model”). We then fit these simulated data with the “full” model that was applied to our empirical data. The number of trials in each simulation was set that obtained in Experiment 1. The full details of the simulation are presented in Appendix A. The simulation results, shown in Fig. A1, demonstrated that when the rate model generated the data, ISR was predominantly accounted for as a change in the accumulation rate. Likewise, when the criterion model generated the data, ISR was predominantly accounted for as an increase in the evidence criterion.

As stated earlier, the accumulation rate is thought to reflect the efficacy with which information is extracted from the stimulus (Ratcliff & Rouder, 1998; Reddi et al., 2003; Roitman & Shadlen, 2002), whereas the evidence criterion may be interpreted in terms of the prior probability that a specific movement is required. Intuitively, it appears plausible to view ISR as linked to the amount of prior belief an observer has in the return location becoming relevant. From this perspective, our finding that ISR is mediated by a change in the accumulation rate is somewhat surprising. In the context of the current experimental design, one plausible interpretation of this finding is that the internal, sensory response to the relevant target change is attenuated at the return location, relative to a non-return location. To test this idea, in Experiment 2 we dissociated the saccade trigger cue from the saccade target location. That is, in one condition the cue appeared at the target location (peripheral cue), as in Experiment 1. In a second condition, a central cue was presented at the currently fixated location which had to be interpreted to determine where to look next (Rafal et al., 1994; Taylor & Klein, 2000). If the inhibitory effect stems from an attenuated sensory response at the return location itself, it should be abolished when the return movement is signalled in the fovea.

## 4 Experiment 2

**Fig. 6:**
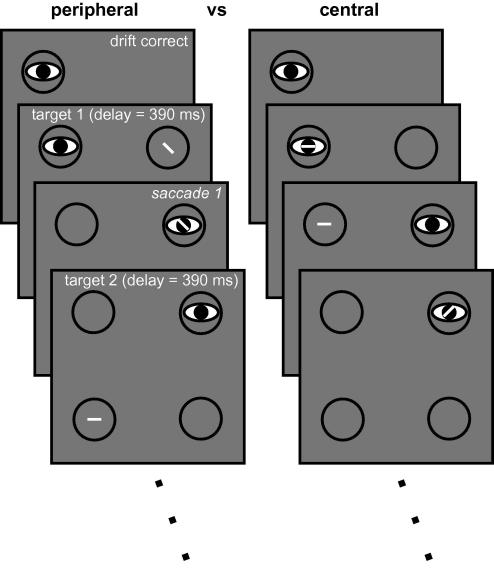
Stimulus design in Experiment 2. The left stream illustrates the peripheral condition in which a small, randomly oriented line segment appeared inside the saccade target. The right stream illustrates the central condition in which the line segment is presented at fixation. The orientation of the line segment, in combination with the current fixation location, signals to the observer where to move to. Only the first two targets are shown, but the whole sequence involved three targets, as in Experiment 1.

The two different cue types used in this experiment are illustrated in Fig. 6, for the first two targets only. The appearance of the cue was kept constant between the peripheral and central conditions, with only the location changing. The rings themselves were always dark (6.7 cd/m^2^) and the cue appeared in the centre of a ring. The cue was a small, bright (142.4 cd/m^2^), oriented line segment. In the peripheral condition, the line appeared inside the target ring itself. Its orientation was randomly chosen between one of: the two diagonal orientations, horizontal and vertical, with the orientation being irrelevant for the task. Accordingly, as in Experiment 1 there was a direct stimulus–response mapping between the cue location and saccade target. In the ‘central’ condition of Experiment 2 this direct mapping was abolished. The small, bright line segment now appeared in the currently fixated ring. The orientation of the line, in combination with the location of the currently fixated ring, determined the next saccade target. For instance, in Fig. 6 the observer starts off fixating the top left ring. A horizontal line segment presented in the fovea now signals that a movement to the top right ring is called for. Once the observer has made the required saccade and is looking at the top right ring, a diagonal line segment signals a movement to the lower left ring. The same five movement sequences were cued in this way as in Experiment 1.

Each observer completed three sessions of both conditions (six sessions in total, run on different days). Half the observers started with three sessions of the peripheral cue condition and then completed the three central cue sessions. The reverse applied for the remaining three observers. We chose to test observers more extensively because of potential concerns about the stability of model parameters with the relatively small distributions of Experiment 1 (see above and Appendix A). One other change from Experiment 1 was the presentation of auditory feedback after each saccade sequence as a motivational aid, in light of the more prolonged testing duration. A 100 ms pure high (750 Hz) or low (500 Hz) tone was played to indicate a correct or incorrect sequence, respectively.

### 4.1 Results

**Fig. 7:**
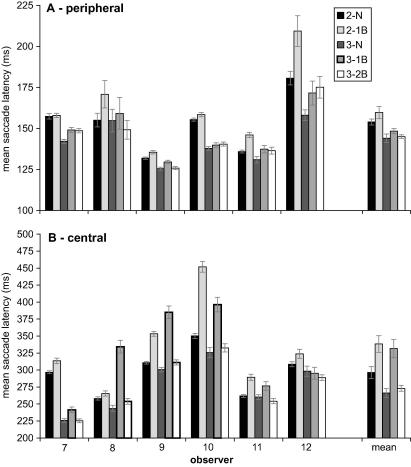
Mean saccade latency for individual observers and the average across observers, in the peripheral (panel A) and central (panel B) conditions of Experiment 2. Conventions are as in Fig. 3.

Fig. 7 illustrates the mean latencies for all six individual observers in both the peripheral (panel A) and central (panel B) conditions. After excluding anticipatory movements, the number of saccades analysed for any one type of sequence per participant ranged from 163 to 180. For the peripheral condition, the data were similar to that of Experiment 1, despite the slight difference in the exact appearance of the peripheral cue. Overall latency appeared somewhat shorter compared to Experiment 1, but this trend can be attributed to a general speeding up with practice over the course of three sessions. The repeated-measures ANOVA on the 1-back data indicated that the 1-back effect was reliable, *F*(1, 5) = 8.44, *p* < .05, as was the effect of saccade number, *F*(1, 5) = 11.56, *p* < .05. The interaction between these two factors was not significant. A pairwise comparison to test for a 2-back effect was not significant, *t*(5) = 1.42.

In the central condition overall latencies were approximately twice as long as those observed in the peripheral condition. This increase in overall latency is not surprising given the relatively arbitrary stimulus–response mapping in this condition and the time needed to interpret the symbolic central cue. A second contributing factor to the latency increase is likely to be the foveal stimulation from the cue itself (Walker, Deubel, Schneider, & Findlay, 1997). The overall magnitude of the 1-back effect was ∼40 ms, averaged across observers and second and third saccades. This 1-back effect was statistically reliable, *F*(1, 5) = 10.25, *p* < .05. There was no main effect of saccade number or interaction between saccade number and ISR. Again, we observed very little evidence for a 2-back effect, *t*(5) = 0.57. Accuracy data are presented in Table 2. Perhaps not surprisingly, accuracy was somewhat lower in the central condition compared to peripheral cueing. There is a hint of an ISR effect in that observers made slightly more errors when the target appeared in the return location.

The average model fits are again shown in Fig. 4. The middle row illustrates the fit to the distributions from the peripheral cueing condition. These plots underline the generally small 1-back effect shown in Fig. 7. The model fits these data well and predicts accuracy close to ceiling, as was observed empirically. For the central condition the model captures the latency data reasonably well and, importantly, the ISR effect in the accuracy.

Model parameters are shown in Fig. 5B (peripheral) and Fig. 5C (central). Not surprisingly, the data from the peripheral condition were not very diagnostic. The ISR effect was greater for second saccades and this effect appears to be mediated through a change in accumulation rate. The average return threshold fell within 1 SEM of the fixed non-return threshold. The small effect in the third saccades does not provide strong evidence for either mechanism. Only the main effect of saccade number was marginally significant, *F*(1, 5) = 5.08, *p* = .07, caused by a general increase in accumulation rate for the third saccades.

The pattern was very clear-cut for the central condition, however. The large behavioural ISR effect was best accounted for as a reduction in the accumulation rate for return targets. The main effect of return location was highly significant, *F*(1, 5) = 18.52, *p* < .01, along with a marginal effect of saccade number, *F*(1, 5) = 4.83, *p* = .08. Again, there was little evidence for a change in the evidence criterion. If anything, the criterion for return saccades is actually reduced (i.e., *opposite* to what would be expected if it was the mechanism causing ISR).

### 4.2 Discussion

The results from Experiment 2 replicated those of Experiment 1 almost exactly, and extended them in important ways. In the peripheral condition we obtained a reliable 1-back effect, but no evidence in favour of 2-back ISR. The overall magnitude of the effect was somewhat reduced compared to Experiment 1. In a central condition the cue and target locations were decoupled. Overall latencies were much prolonged under these circumstances and the 1-back effect was very pronounced. Again, we found little evidence for the existence of a 2-back effect in this condition. In the central cueing condition, ISR was consistently accounted for as a change in the mean rate of accumulation, with a lower rate for return compared to non-return saccades. The finding that ISR is accounted for by a rate change even when the movement cue is presented away from the return location itself suggests it is not necessarily the *sensory* response to events at the return location that results in the slower accumulation of evidence. A similar point was suggested by Rafal et al. (1994), who found saccadic slowing in a standard cue-target IOR paradigm with the required response being a saccade directed by a symbolic cue (an arrow; see also Taylor & Klein, 2000). This is consistent with the general conclusion that inhibition is not wholly due to a slower of extraction of information from a previously fixated or attended location. We return to this point below.

## 5 General discussion

The latency of saccades that return gaze to a location that was fixated previously is prolonged relative to saccades that bring the line of sight onto a “new” location. We devised a novel paradigm to experimentally control sequences of three saccadic eye movements. This paradigm allowed us to examine ISR for both peripheral and central (symbolic) cues. Our empirical data suggest that ISR is a reliable phenomenon, even at the level of individual observers. The effect was more pronounced for saccades that were triggered by a central cue compared to peripheral cues presented at the saccade target location, and these data were particularly diagnostic in the identification of the underlying mechanism. At this stage, we cannot identify whether this finding is due to different systems being involved in the programming of more exogenous versus endogenous saccades (Schiller, 1998), or to a scaling of ISR with overall response latency. In this particular paradigm ISR only extended back to the immediately preceding fixation location, although there were clearly some individual observers for whom ISR also occurred for the location fixated two saccades ago.

We modelled saccade latency distributions with the LBA model (Brown & Heathcote, 2008), which assumes a single accumulator for each response alternative. The accumulators are subject to independent, random variation in their rate of rise and in the starting point of accumulation. This model contains two mechanisms that allow it to produce a difference between a pair of latency distributions: a change in the mean accumulation rate, and in the amount of evidence that needs to be accumulated before a response is initiated (see Fig. 1). Both mechanisms also naturally predict corresponding changes in choice probability: the chances of one of the competing accumulators winning the race to threshold are increased if the accumulation rate associated with the target is reduced, or its evidence criterion increased. The model was fit to the second and third saccade 1-back data for each individual observer. Where ISR was prominent in the data, it was consistently accounted for as a change in the rate of accumulation. In none of our experiments did we obtain any evidence for an increase in the evidence criterion^1^. It is fair to say that the strong evidence in favour of a rate change was found under the specific assumption of linear ballistic accumulation. However, it is well-known that different models based on the sequential sampling of evidence can be difficult to distinguish from each other on the basis of behavioural data (Ratcliff & Smith, 2004). As such, our general assumption is that where the evidence in favour of a rate change is clear, the corresponding mechanism will be at play in alternative models from this family (e.g., leaky competitive integration; Usher & McClelland, 2001).

At this stage, it is worth pointing out that we have restricted our analyses to just two possible underlying mechanisms of ISR. This choice was based on the rich tradition of accounting for choice and RT data in the framework of evidence accumulation. In psychology one is typically trying to account for latency differences that arise from the information processing demands of certain experimental conditions. As such, the parameters of interest are typically those that are thought to reflect these demands (e.g., extracting evidence, speed-accuracy settings, etc.). However, it is of course possible that distributional differences may be accounted for through other model components. In particular, one might have expected ISR to be mediated by the non-decisional component of the saccade latency, *T_er_* (this could, for example, reflect a longer peripheral motor delay for direction reversals; Hooge & Frens, 2000). Note, however, that a change in the non-decisional component would simply shift the return distribution rightwards, without affecting its shape. Across the different experimental conditions it is clear that the shape of the return distribution does change (see Fig. 4), in that it typically has greater variance and rightward skew compared to the non-return distribution. Initial model fits indeed demonstrated that a non-decisional shift was not a viable competitor for the rate and criterion models.

### 5.1 What is accumulated in accumulator models?

Accumulator models of many different flavours have a long and successful history in accounting for perceptual choice and reaction time data (see reviews by Luce, 1986; Ratcliff & Smith, 2004). In the introduction we reviewed some of the evidence that suggests that the brain indeed implements the decision mechanisms posited by this class of models. Both behavioural and neurophysiological evidence suggest that the accumulation rate is affected by the stimulus quality and that the evidence criterion is modulated by the prior probability of a particular response.

We entertained the idea that an attenuated sensory response to stimulation at a recently fixated location might be responsible for a lower accumulation rate for a response to that location. Indeed, this would be a plausible explanation for a reduced accumulation rate under peripheral cueing conditions. However, such an account does not hold for the data from the central cueing condition of Experiment 2 (see also Rafal et al., 1994; Taylor & Klein, 2000). In this condition the cue location was dissociated from that of the saccade target. As such, reduced visual processing at the target location should not have affected the sensory response to the cue.

Therefore, it seems unlikely that sensory evidence alone is what drives the rate of accumulation to threshold. A wider interpretation is that sensory evidence is one of a multitude of possible contributors to the overall “desirability” of a particular course of action (Dorris & Glimcher, 2004). In the behavioural domain for instance, word frequency effects in lexical decision tasks have been accommodated in the accumulation, or drift, rate (Ratcliff, Gomez, & McKoon, 2004). Such effects are clearly not of a sensory nature, but rather reflect learned associations which affect the efficacy with which information is extracted from the stimulus.

Thus, one hypothesis is that a more inclusive representation of desirability of a motor response is accumulated by the decision-making system. We speculate that the reduced accumulation rate of return saccades may reflect the system having learned, over the course of its lifetime, that it is highly unlikely that a recently inspected location turns out to be behaviourally relevant in the next instant (Anderson et al., 2008). As such, immediate re-inspection is typically a waste of useful resources that could have been deployed more productively elsewhere. In other words, the temporal structure of the environment within which the system has evolved is such that that re-inspection is less likely to be rewarded with success (e.g., finding a target). As a consequence, the system is “reluctant” to interpret the incoming sensory information as commanding a return movement, whether that information is presented at the return location itself or elsewhere.

### 5.2 ISR and IOR

It is useful to examine the relation between our findings with those reported in the IOR domain. Classic IOR is typically studied in a cueing paradigm. As stated in the introduction, this paradigm is assumed to evoke three attentional shifts (Posner & Cohen, 1984), of which the final one (to the target location) is of critical interest. The “equivalent” comparison in our paradigm would be between sequence types 2 and 3 (see Fig. 2). This contrast was not analysed in detail, because we wanted to compare 1-back and 2-back saccades with a common baseline. The mean ISR effect (across observers) based on these two sequence types was 6 ms (Experiment 1), 0 ms (Experiment 2, exogenous), and 41 ms (Experiment 2, endogenous) ms. These values are somewhat reduced in comparison with the contrast between sequence types 4 and 1 that was used in the modelling above, but follow the same pattern.

One influential account for IOR is predicated on oculomotor programming (Rafal, Calabresi, Brennan, & Sciolto, 1989). The idea is that IOR arises in those situations in which a cue (whether it be exogenous or endogenous) triggers the programming of a saccade to a peripheral (potential) target location. This saccade need not be executed, but its planning is required for IOR to occur (i.e., it may be programmed, but cancelled; cf. Rizzolatti, Riggio, Dascola, & Umilta, 1987). Provided the cue-target interval is sufficiently long, IOR is observed for both manual (Rafal et al., 1989) and saccadic (Rafal et al., 1994; Taylor & Klein, 2000) responses to the target at the cued location. Naturally, this idea is compatible with the very existence of ISR, and is more generally consistent with suggestions that attention and gaze shifts have a common neural basis (Kustov & Robinson, 1996; Rizzolatti, Riggio, & Sheliga, 1994; Rizzolatti et al., 1987). The oculomotor hypothesis also offers to explain why IOR is only observed for peripheral cues when the eyes remain fixed: under such circumstances, peripheral (but not central) cues automatically elicit the programming of saccades to those locations.

A study by Taylor and Klein (2000) deserves special mention regarding the role of saccadic programming in IOR. These authors comprehensively tested the existence of IOR under conditions in which central and peripheral cues elicited no, a manual, or a saccadic response, followed by either a manual or saccadic response to the target. Of particular interest is their ‘saccadic–saccadic’ condition. In this condition, participants generated a saccade to a peripheral location on the basis of a peripheral or central (arrow) cue. After 500 ms a tone signalled they should return gaze to the central fixation point. Subsequently a peripheral target or central arrow demanded the final, and critical, saccadic response to the target box. These conditions approximate those of our studies very closely, and indeed a robust IOR effect was found for all four combinations of peripheral and central cues and target signals. Interestingly, across the full range of cue and target response combinations, the authors reported a dissociation depending on whether, at any stage in the cue – target sequence a saccadic response was required. If so, IOR was observed in response to both central arrows and peripheral targets; however, if only manual responses were produced (to both the cue and the target, or to just the target) whilst the eyes remain fixed in the centre at all times, only peripheral targets triggered IOR (see also Rafal et al., 1994). This dissociation was accounted for by assuming that when the eyes remain fixed, IOR in (relatively arbitrary) manual responses reflects inhibited attention and/or perception (see also Ivanoff & Klein, 2006). In contrast, when the eyes are free to move IOR is taken to reflect a general motor bias against responding in the previously cued direction. In accordance with the oculomotor hypothesis of Rafal et al. (1989), Taylor and Klein (2000) speculated that in addition to *revealing* the effects of IOR, saccadic responses also *produce* IOR (even if the subsequent response is manual).

This notion that IOR may have either an attentional-perceptual or a motoric cause is pervasive throughout the IOR literature (Taylor & Klein, 1998). The motor bias that Taylor and Klein (2000) evoked to account for IOR when saccadic responses are involved is often portrayed as a criterion shift, analogous to the idea of bias in a signal detection framework (Ivanoff & Klein, 2004, 2006; Taylor, 2007). A criterion shift appears to, at first sight, directly map onto the evidence criterion in the modelling carried out here. However, we would caution against a direct mapping between the motor bias hypothesis and the evidence criterion parameter in our model. Our conception of the decision unit is inherently as a motor structure. That is, in our view the target-related accumulator codes a movement vector to a specific location in the visual field. Such codes are found throughout the oculomotor system (Wurtz & Goldberg, 1989). As discussed in the previous section, the reduction in accumulation rate within the decision unit may stem from a variety of sources that together determine the overall desirability of a particular movement vector. In fact, we argued against a purely sensory interpretation of our findings on the basis that the relevant saccade trigger in the central condition of Experiment 2 was de-coupled from the saccade target location itself. Thus, our finding that ISR is best accounted for by a change in accumulation rate should not be taken as evidence in favour of a perceptual account of the inhibitory effect.

Finally, recent studies have begun to probe the neural basis for IOR. When monkeys generate a sequence of three saccades, with the third saccade directed to either the first target location or to the opposite, new, target location, a behavioural advantage for the return location emerges (Dorris, Taylor, Klein, & Munoz, 1999), unlike in humans (Anderson et al., 2008; Carpenter, 2001; Rafal et al., 1994; Taylor & Klein, 2000). One possibility that has been raised to account for this difference is the extensive training received by monkeys in these studies (Fecteau & Munoz, 2003). However, using the standard cueing paradigm in which the monkey makes a single saccade to a peripheral target after having been exogenously cued, IOR is reliably observed (Bell, Fecteau, & Munoz, 2004; Dorris et al., 1999).

These studies show that visuo-motor cells within the intermediate layers of the SC show an attenuated stimulus-related response to a target in a cued location. In addition, it appears that the subsequent build-up of activity towards the saccade trigger threshold takes place at a slower rate, but the threshold itself remains fixed; this is exactly what was observed in our modelling of ISR. Dorris, Klein, Everling, and Munoz (2002) found no evidence that IOR acts to suppress collicular baseline activity in response to a cue. In fact, baseline activity in cells responsive to the cued location was somewhat elevated. Interestingly, the authors conclude that IOR is not generated at the collicular level, but rather reflects the attenuated inputs received from upstream areas. In their study, the attenuated input was likely to be the reduced visual response to the target stimulus itself. The modelling of our behavioural data strongly suggests that the speed with which activity accumulates towards the production of a saccadic response, is also governed by more psychological, non-sensory inputs. More generally then, our results support the notion that target selection is served by a representation of salience that includes a multitude of converging signals (Fecteau & Munoz, 2006).

## Figures and Tables

**Table 1:** Overview of model parameters.

Parameter	Meaning	Constraints
*v_T_*	Mean accumulation rate for the target	>0
*v_NT_*	Mean accumulation rate for non-targets	>0
*σ_v_*	Accumulation rate standard deviation, common to all accumulators	>0
*s*	Upper limit of the uniform starting point variability, common to all accumulators	*z* > *s* ⩾ 0
*z*	Response threshold associated with non-return locations (fixed to 1)	1
*α*	Proportional adjustment of the mean accumulation rate associated with a return location	>0
*β*	Proportional adjustment of the threshold associated with a return location	>0
*T_er_*	Constant, non-decisional component of the latency	50 ⩾ *T_er_* ⩾ min latency

**Table 2:** Mean proportion of saccades landing in the target acceptance region. The absolute deviation from the model prediction is given in parentheses (for non-return and 1-back saccades only).

Experiment	2-N	2-1B	3-N	3-1B	3-2B
1	.94 (.01)	.93 (.02)	.96 (.02)	.94 (0)	.95
2 – Peripheral	.99 (0)	.98 (0)	.99 (0)	.98 (0)	.98
2 – Central	.96 (0)	.92 (0)	.91 (.02)	.86 (.02)	.93

## Appendix A

The full model has two mechanisms to account for a difference between a pair of latency distributions: a change in the accumulation rate and a change in the evidence criterion. These changes can effectively be considered as two distinct, nested versions of the full model in which both parameters are allowed to vary. That is, the nested rate model may be regarded as the full model, but with *β* = 1. Likewise, the nested criterion model is the full model with *α* = 1. The critical issue we address here is whether data generated by one or the other nested model are successfully classified as such in a fit of the full model in which both mechanisms are free to vary. This issue is particularly important in the context of Experiment 1 in which the number of observations per latency distribution was modest and the magnitude of ISR was relatively subtle (∼18 ms across second and third saccades).

The simulation strategy was as follows:

1. For each observer, use their best fitting nested model parameters to simulate 60 return and non-return trials (the same number that the human participants were tested on in Experiment 1).
2. Note the number of simulated errors and compute the defective latency distributions for both return and non-return saccades, using the same quantiles as in the actual experiments.
3. Fit the latency and accuracy data with the full seven-parameter model, as described in the main text.
4. Perform steps 1–3 *M* times (*M* = 100), to create a total of 600 simulated data sets.
5. Perform steps 1–4 separately for second and third saccade data (recall that ISR was more pronounced for third saccades), and separately for the rate and criterion generating models.

Fig. A1 shows the mean values of the critical model parameters that can reflect ISR. The *α* and *β* parameters (see Table 1 in the main text) adjust the accumulation rate and criterion, respectively, to account for ISR. Their values correspond to the return/non-return ratio of the relevant parameter. The mean values of these ratios are shown in Fig. A1 (for second and third saccades in the left and centre panels, respectively).

The horizontal dashed lines in these plots indicate the expected value if a particular mechanism was not involved in generating ISR. It is clear that, for both second and third saccades, when the rate model generated ISR the mean accumulation rate was lower for return saccades compared to non-return saccades. In contrast, the return threshold was close to 1. The reverse was true when the criterion model generated the data: the return threshold was elevated with the mean accumulation rate lying close to 1. However, note that there is a very slight tendency for data generated by the rate model to be accounted for through a change in the evidence criterion, as evidenced by the somewhat elevated thresholds. This is evidence that mis-classification does occur at the level of individual data sets and that this occurs with sufficient frequency to elevate the overall mean threshold estimate. The implication is that it is actually slightly more difficult to find evidence in favour of the rate model compared to the criterion model, adding strength to the evidence found in our empirical data in favour of the rate model. A repeat of the third saccade simulation with a greater number of trials demonstrates, unsurprisingly, improved parameter identifiability (Fig. A1 – right panel).

In conclusion, for these simulations in which we “know” the true causal mechanism of ISR, the full model, on average, reduced to a nested version that matched the generating model, particularly for the larger number of simulated trials. This is of course exactly the desired outcome if we wish to use the parameters of the full model fits to identify the mechanism of ISR. The added value of the full model is that it can, in principle, identify whether both mechanisms contribute and, if so, their relative importance.

## Footnotes

1 An alternative modelling strategy would have been to fit the empirical data with two nested versions of the full model used here. A rate model (six parameters) accounts for ISR only in terms of a change in mean accumulation rate; the evidence criterion is forced to be the same for return and non-return saccades. A criterion model (six parameters) accounts for ISR through an increase in the evidence criterion for return saccades, but does not allow for a change in mean accumulation rate. Given the equal number of free parameters of these restricted models, a straight comparison of their log-likelihoods elucidates which mechanism provides the better account for the experimental phenomenon under consideration. We computed the log-likelihood difference between the fits of the rate and criterion models, for each individual observer, and for both second and third saccades. Summed across observers, these differences were 2.8, 4.8, and 38.9 for Experiment 1, Experiment 2 – peripheral, and Experiment 2 – central, respectively. A positive log-likelihood difference in this instance corresponds to evidence in favour of the rate model. To put these numbers into perspective, a log-likelihood difference of 2.8 corresponds to a likelihood-ratio of ∼16. On the whole then, the results from this analysis corroborate the direct parameter estimates from the full model, with the rate model being particularly well supported by the results from the central condition of Experiment 2.
